# Proteomic Analysis Reveals the Phenotypic Heterogeneity and Tolerance Mechanisms of Halophilic *Vibrio parahaemolyticus* Under Dual Stress of Low Salinity and Bile Salts in the Human Intestine

**DOI:** 10.3390/biom15040518

**Published:** 2025-04-01

**Authors:** Yingying Guo, Bing Yang, Xiaoyan Zhou, Zhangxi Gong, Enxiao Wang, Yingjie Pan, Yong Zhao, Haiquan Liu

**Affiliations:** 1 College of Food Science and Technology, Shanghai Ocean University, Shanghai 201306, China; m220300897@st.shou.edu.cn (Y.G.); m220351109@st.shou.edu.cn (X.Z.); m230351085@st.shou.edu.cn (Z.G.); m220300944@st.shou.edu.cn (E.W.); yjpan@shou.edu.cn (Y.P.); 2 Shanghai Majorbio Bio-Pharm Technology Co., Ltd., Shanghai 201318, China; bing.yang@majorbio.com; 3 Shanghai Engineering Research Center of Aquatic-Product Processing & Preservation, Shanghai 201306, China; 4 Laboratory of Quality & Safety Risk Assessment for Aquatic Product on Storage and Preservation (Shanghai), Ministry of Agriculture and Rural Affairs, Shanghai 201306, China; 5 Engineering Research Center of Food Thermal-Processing Technology, Shanghai Ocean University, Shanghai 201306, China; 6 Food Industry Chain Ecological Recycling Research Institute, Food Science and Technology College, Shanghai Ocean University, Shanghai 201306, China

**Keywords:** *Vibrio parahaemolyticus*, proteomics analysis, phenotypic heterogeneity, tolerance mechanisms, low salinity, bile salts

## Abstract

*Vibrio parahaemolyticus*, a halophilic Gram-negative bacterium commonly found in aquatic products, can colonize the human small intestine, causing gastroenteritis and potentially leukemia. As a major intestinal pathogen, it poses a significant threat to public health. This study aims to investigate the phenotypic heterogeneity of *V. parahaemolyticus* in the low-salinity and bile salt environments of the human intestinal tract and to elucidate its mechanisms of tolerance and pathogenicity using proteomics. The experimental results indicated that under the low salinity and bile salts conditions of the human intestinal environment, the growth, motility, and biofilm formation of the strains were significantly inhibited. Proteomics analysis revealed that, under these conditions, the energy metabolism, chemotaxis system, flagellar motor, and ribosome-related proteins of *V. parahaemolyticus* were significantly affected, thereby influencing its growth, motility, and biofilm formation. Furthermore, the activation of the secretion system, particularly the T2SS, enhanced the virulence of secreted factors on host cells. Additionally, the activation of the β-lactam resistance pathway increased resistance to the intestinal environment, thereby enhancing the pathogenicity of *V. parahaemolyticus*.

## 1. Introduction

*Vibrio parahaemolyticus* is a halophilic Gram-negative bacterium first identified during a food poisoning outbreak in Osaka, Japan, in 1950 [1]. In the presence of bile acids in the gut, it can activate various virulence factors (e.g., adhesion factors, lipopolysaccharides, hemolysins, type III and type VI secretion systems, iron uptake systems, proteases, and outer membrane proteins), leading to watery diarrhea, vomiting, severe dehydration, and even death [2]. Over the past 25 years, it has emerged as the leading cause of foodborne illness and infectious diarrhea in coastal countries worldwide [3].

Bile salts work as part of lipid emulsification in the human intestine and exhibit strong antibacterial activity against pathogenic bacteria. However, intestinal bacteria can adapt to recognize bile salts, respond to signals, and induce the production of virulence factors, thereby enhancing their survival within the host [4]. *V. parahaemolyticus* typically enters the body through contaminated water or food, survives, and colonizes the small intestine. Many researchers are investigating the antimicrobial properties of bile salts and their role in pathogenic mechanisms. For example, the VtrA/VtrC complex and VtrB respond to bile salts, activate the type III secretion system (T3SS), and inject virulence factors into host cells, thereby enhancing cytotoxicity in *V. parahaemolyticus* [5]. *V. parahaemolyticus* enhances virulence under bile acid stress by regulating membrane transport, RND efflux pumps, and secretion systems, thereby causing AHPND in white shrimp (*Litopenaeus vannamei*) [6]. In *Vibrio cholerae*, ToxR/S senses the bile salts environment, induces bile acids resistance, and activates the expression of virulence factors [7]. Interestingly, nearly all current studies on the adaptive mechanisms of *V. parahaemolyticus* under bile salts stress and its interactions with host cells have been conducted under optimal high-salinity conditions [8,9].

However, when strains actually enter the human body and colonize the gut, they experience dual stress from low salinity and bile salts. Meanwhile, several studies have shown that salinity significantly affects the phenotype and pathogenicity of *V. parahaemolyticus* [10]. Zhang [11] found that low salinity environments can also activate the type III secretion systems 1 and 2 (T3SS1 and T3SS2) in *V. parahaemolyticus*, mediating cytotoxicity to HeLa cells and affecting biofilm formation and c-di-GMP production [12]. Concurrently, preliminary experiments showed that strains originally grown under 0.20% bile salts could not grow when the salinity was reduced to 0.90%. It can clearly be seen that salinity is also a crucial factor in understanding the pathogenic mechanisms of bacteria in the human body. Thus, the phenotype of *V. parahaemolyticus* after bile salts stimulation in a high-salinity environment does not accurately reflect its state after entering the human intestinal environment. Moreover, the molecular mechanisms explored through histological analyses also failed to accurately reflect the mechanisms underlying *V. parahaemolyticus* tolerance to the human bile salts environment and its pathogenesis.

Thermostable direct hemolysin (TDH), TDH-related hemolysin (TRH), and thermolabile hemolysin (TLH), encoded by *tdh*, *trh,* and *tlh* genes, respectively, are closely related to its pathogenicity [13]. Thereby, in this study, four strains containing different virulence genes were selected as research objects. Salinity was controlled at 0.90%, while bile salt concentrations were varied to comprehensively analyze the phenotypic heterogeneity of *V. parahaemolyticus* in terms of growth, motility, and biofilm formation under different salinity conditions. This approach aimed to simulate the actual conditions that pathogens encounter in the human intestine. Additionally, proteomics was used to elucidate the phenotypic heterogeneity and molecular mechanisms of tolerance to low salinity and bile salts in the intestinal tract. This study provides a theoretical basis for further research on antimicrobial agents targeting foodborne pathogens.

## 2. Materials and Methods

### 2.1. Strains, Cell Lines, and Culture Conditions

Four strains (VP17, VP44, VP49, VP85) containing different virulence genes were used as test strains in this study. These strains were isolated from the feces of patients with acute diarrhea (Table 1). Bacteria stored at −80 °C was selected and cultured on TCBS agar (Beijing Land Bridge Technology Co., Ltd., Beijing, China). Single colonies of each strain were inoculated into 5 mL of 3.00% NaCl (wt/vol) (TSB + N) broth (Beijing Land Bridge Technology Co., Ltd., Beijing, China) and cultured to the logarithmic phase. This process of bacterial activation twice puts the bacteria in an optimal state. The THP-1 and Caco-2 cell lines (Beyotime Biotechnology Co., Ltd., Shanghai, China) were maintained in RPMI 1640 and DMEM, respectively (Gibco, Waltham, MA, USA) (Table 1). Both cell lines were supplemented with 10% fetal bovine serum (FBS) (Gibco, Waltham, MA, USA ) and 1% penicillin–streptomycin (Gibco, Waltham, MA, USA) and cultured in a humidified atmosphere containing 5% CO_2_. All bacterial strains and cell lines were cultured at 37 °C.

### 2.2. Preparation of Media with Different Salinities

Based on the bile salt concentration range of 0.03–0.3% (*w*/*v*) in the human small intestine, low salinity media with different bile salt concentrations were prepared using tryptone, yeast extract, NaCl, and human bile salts [14]. The concentrations were as follows: 3.00% NaCl (*w*/*v*), 0.90% NaCl (*w*/*v*), 0.87% NaCl (*w*/*v*) + 0.03% bile salts, 0.85% NaCl (*w*/*v*) + 0.05% bile salts, 0.80% NaCl (*w*/*v*) + 0.10% bile salts, 0.75% NaCl (*w*/*v*) + 0.15% bile salts, 0.70% NaCl (*w*/*v*) + 0.20% bile salts, and 0.60% NaCl (*w*/*v*) + 0.30% bile salts. Bacterial swimming plates were prepared by adding 0.30% agar to the media with different bile salt concentrations [15].

### 2.3. Bacterial Growth Curve Determination

After two times of activation, bacteria were cultured to the logarithmic phase and inoculated into media with different bile salt concentrations (the initial bacterial quantity in broth was about 10^4^ CFU/mL). The OD_600nm_ values were measured every 30 min to determine the growth curve of the strains, and each experiment was performed in triplicate (Oy Growth Curves Ab Ltd., Raisio, Finland). Growth kinetics were fitted using a modified Gompertz model for *V. parahaemolyticus* growth data at different bile salt concentrations and fitted by Origin software [16].

y = A + B *exp*{−*exp* [*μ_max_* (C − x)/A + 1]}, (1)

In this equation:

A represents the initial inoculum of the strains (CFU/mL);

B represents the difference between the maximum bacterial concentration and the initial bacterial concentration (CFU/mL);

*μ_max_* represents the maximum specific growth rate;

C represents the lag time (LT), the time at which bacterial growth is detected (h);

x represents the time;

y represents the total number of bacteria at that time (CFU/mL).

### 2.4. Bacterial Motility Assay

Vertical drops of 5 μL of logarithmic phase bacterial suspension (~10^8^ CFU/mL) were added to swimming plates containing different bile salts concentrations. After 24 h, the motility diameters of the four strains were measured [15].

### 2.5. Measurement of Biofilm Production

Biofilm production by strains at different salinities was quantified using the crystal violet staining method. Moreover, 10 μL of logarithmic-phase *V. parahaemolyticus* suspension and (~10^8^ CFU/mL) and 990 μL of medium containing different bile salt concentrations were added to 24-well plates. The strains were cultured at 37 °C for 12, 24, 36, 48, and 60 h, and biofilm formation was quantified using crystal violet staining. The biofilms were gently washed three times with 0.01 M PBS, stained with 1 mL of 0.1% crystal violet at 37 °C for 30 min, and then dissolved in 1 mL of 95% ethanol for 30 min. The optical density (OD_600nm_) of each well was measured. Non-inoculated TSB wells served as blank controls, and six replicates were used for each strain [17].

### 2.6. Confocal Laser Scanning Microscope (CLSM) Observation of Biofilm Structure

Biofilms were formed directly on confocal dishes according to the above method and incubated at 37 °C until the maximum biofilm formation was observed by CLSM (Leica TCS SP8, Wetzlar, Germany). The suspended bacterial liquid was discarded, and the dishes were washed with 0.01 M PBS, air-dried, and then treated with 1 mL of 4% glutaraldehyde solution for 30 min at 4 °C. The dishes were washed with 0.01 M PBS (2–3 times) to remove excess glutaraldehyde, and then stained with 200 μL of SYBR Green I dye in the dark at 25 °C for 30 min. After rinsing with PBS and drying, the biofilm structure was observed using CLSM (40× objective, 488 nm excitation wavelength, 500–550 nm emission wavelength) [18].

### 2.7. CLSM for Cell Membrane Damage Study

Four strains were secondarily activated (~10^8^ CFU/mL) and treated at different salinities for 12–18 h. Bacterial suspensions were then stained according to the instructions of the manufacturer of the Bacterial Viability/Cytotoxicity Assay Kit (Sangon Biotech, Co., Ltd., Beijing, China). The excitation/emission wavelengths for NucGreen and EthD-III were 503/530 nm and 530/620 nm, respectively. Confocal images were obtained using CLSM (Leica TCS SP8, Wetzlar, Germany) at 40× magnification. The green color (NucGreen) was excited at 488 nm and emitted at 510–610 nm. The red color (EthD) was excited at 552 nm and emitted at 571–630 nm.

### 2.8. Scanning Electron Microscopy (SEM) for Cell Membrane Damage Study

Sterile silicon slides (1 cm × 1 cm × 0.6 mm) were placed in 24-well plates containing 1 mL of medium with different bile salt concentrations. *V. parahaemolyticus* was inoculated into the 24-well plates and incubated at 37 °C. After incubation, the slides were washed twice with PBS (pH 7.2, 0.01 M) to remove planktonic cells and then fixed with 2.5% glutaraldehyde (Damao chemical reagent factory, Tianjin, China) at 4 °C for 12 h. Biofilm dehydration was performed using ethanol solutions of 30%, 50%, 70%, 90%, 95%, and 100% for 15 min each (Damao chemical reagent factory, Tianjin, China). After drying, the biofilms were coated with gold and examined using a scanning electron microscope (SEM; S-4800, Hitachi, Tokyo, Japan) [19].

### 2.9. Proteomic Analysis of VP17 Under Different Salinity Stresses

To investigate the tolerance mechanisms of *V. parahaemolyticus* under the dual stress of low salinity and bile salts in the human intestine, VP17 (the most abundant tdh^+^/trh^−^ genotype in human isolates) was selected for proteomic analysis [20]. After secondary activation, VP17 was added to the media with different salinities and cultured to the logarithmic phase, and centrifugation yielded the organisms for proteomic sequencing, comparison with several databases (NR, Swiss-Prot, Pfam, EggNOG, GO, and KEGG), and protein function annotation.

### 2.10. Intracellular ROS of V. parahaemolyticus Under Different Salinity Stresses

The secondary activated VP17 (~10^8^ CFU/mL) was treated at different salinities for 12 h–18 h, respectively. Centrifuge at 12,000 rpm for 2 min to remove the medium; wash 3 times with 0.01 M PBS. Resuspend the strains in the medium diluted with 10 μM DCFH-DA (Beyotime Biotechnology Co., Ltd., Shanghai, China), incubate at 37 °C for 20 min, and invert and mix every 3–5 min to ensure full contact between the probe and the strains. After centrifugation, wash 3 times with media of different salinities to thoroughly remove DCFH-DA that had not entered the strains. Since the fluorescence spectrum of DCFH was very similar to that of FITC, the FITC channel was selected on the flow cytometer (BD Accuri C6, Franklin Lakes, NJ, USA) to detect intracellular ROS in bacteria.

### 2.11. CCK-8 Assay for Cytostatic Rate

To determine the pathogenicity of *V. parahaemolyticus* under different salinity stresses, intestinal epithelial cells (Caco-2) and macrophages (induced from THP-1) were used as cell models. These cells, which are the body’s first line of defense against pathogenic microorganisms, were used to assess the inhibitory effects of *V. parahaemolyticus* using the Trans Detect Cell Counting Kit-8 (Sangon Biotech, Shanghai, China). After the second activation, an equal amount of bacteria was added to media with different salinities for 2 h, then centrifuged at 4 °C, 12,000 rpm for 10 min. The supernatant was filtered through a 0.22 μm filter membrane to remove bacteria, and the sterile filtrate was collected. One hundred microliters of cell suspension (1 × 10^5^ cells) was added to each well of a 96-well plate and induced with 80 ng/mL of PMA (Merck Chemicals & Technologies Ltd., Shanghai, China) for 24 h. Ten microliters of sterile filtrate was added to each well, and the plate was incubated in a cell culture incubator for 24 h [21]. Add 1:10 (*v*/*v*) premixed complete culture medium with CCK-8 solution, incubate at 37 °C, 5% CO_2_ for 30 min, and measure the absorbance at 450 nm [22]. The inhibition rate (C) of the cells was calculated according to the following formula.

C (%) = 100 × (A1 − A0)/(A1/A2),(2)

In this equation, the following definitions apply:

A0 represents the absorbance of experimental wells (cells infiltrated by toxin);

A1 represents the absorbance of control wells (containing cells, medium, and CCK-8 wells);

A2 represents the absorbance of blank wells (containing medium and CCK-8 wells).

### 2.12. Visualization of the Molecular Docking Between Efflux Pump Proteins and Bile Salts Molecules

Docking calculations were conducted with AutoDock 4.2, downloading the protein structure from the PDB, adding hydrogen, and removing water molecules and ligands to the protein structure converting the prepared protein into a molecularly docked receptor. Draw the bile salts molecule using ChemDraw 20.0, convert it using Chem3D 20.0, and then add the hydrogen atoms. All possible ligand flexibility options were turned on, while the early termination option was turned off. Every possible form of the bile salts molecule was docked to the protein. Poses with the highest docking scores and reasonable bonding patterns were selected and visualized using PyMOL 1.7.

### 2.13. Data Processing and Statistical Analysis

Growth curve fitting analysis was performed using Origin Pro 2021 software (Origin Lab Corp., Northampton, MA, USA). Data were analyzed and plotted for significant differences using SPSS Statistics 26.0 software (SPSS Inc., Chicago, IL, USA) and GraphPad Prism 8 (Graphpad Software Inc., San Diego, CA, USA).

## 3. Results

### 3.1. Growth

**Figure 1:**
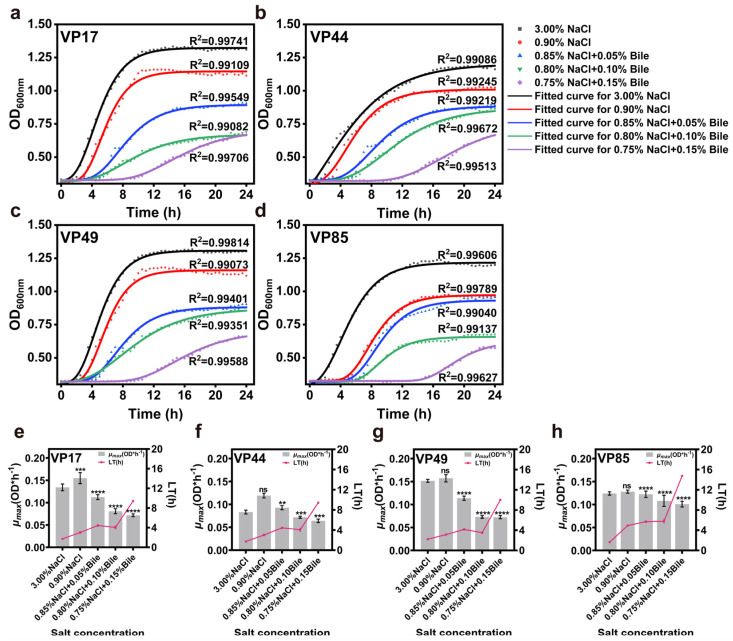
Impact of low salinity and bile salts on the growth of *V. parahaemolyticus*. (**a**–**d**) Growth curves of VP17, VP44, VP49, and VP85 under different salinities. (**e**–**h**) Evaluation of *μ_max_* and LT of VP17, VP44, VP49, and VP85 under different salinities. Significance was compared to the control, ns > 0.05, ** *p* < 0.01, *** *p* < 0.001, **** *p* < 0.0001.

It is well known that bile salts in the human gastrointestinal tract affect the growth and pathogenicity of pathogenic bacteria. To further investigate the growth characteristics of halophilic *V. parahaemolyticus* after entering into the human low salinity and bile salts environment. Based on the physiological range of human bile salts (0.03–0.30% *w*/*v*), a preliminary experiment showed that four strains could not grow at 0.20% bile salts (*w*/*v*). Therefore, the bile salts concentration range was set to 0.05–0.15% (*w*/*v*). The results showed R^2^ > 0.99 for all fitted curves, indicating a better growth kinetic model. When the initial inoculum was consistent, the growth of all strains was inhibited compared to those cultured at 3.00% NaCl. The maximum bacterial concentration decreased significantly with decreasing salinity and increasing bile salts concentration, particularly in the experimental group with added bile salts (Figure 1a–d). It was evident that both total salinity and bile salts concentration significantly influenced the growth of pathogenic bacteria.

Microbial growth is typically divided into four phases: lag phase, logarithmic phase, stationary phase, and decline phase. Changes in the external environment significantly affect bacterial growth rate, lag time (LT), tolerance, and maximum specific growth rate (*µ_max_*), all of which reflect bacterial adaptability to the environment [23]. The results showed that, as the medium salinity decreased and bile salts concentration increased, the LT of all four strains increased. Moreover, when the bile salts concentration reached 0.15%, LT significantly increased, indicating that the strains required a longer adaptation time. When salinity decreased from 3.00% to 0.90%, *µ_max_* of VP17, VP44, VP49, and VP85 increased under low salinity conditions. After the addition of bile salts, *µ_max_* decreased gradually with increasing bile salts concentration (Figure 1e–h).

### 3.2. Motility

**Figure 2:**
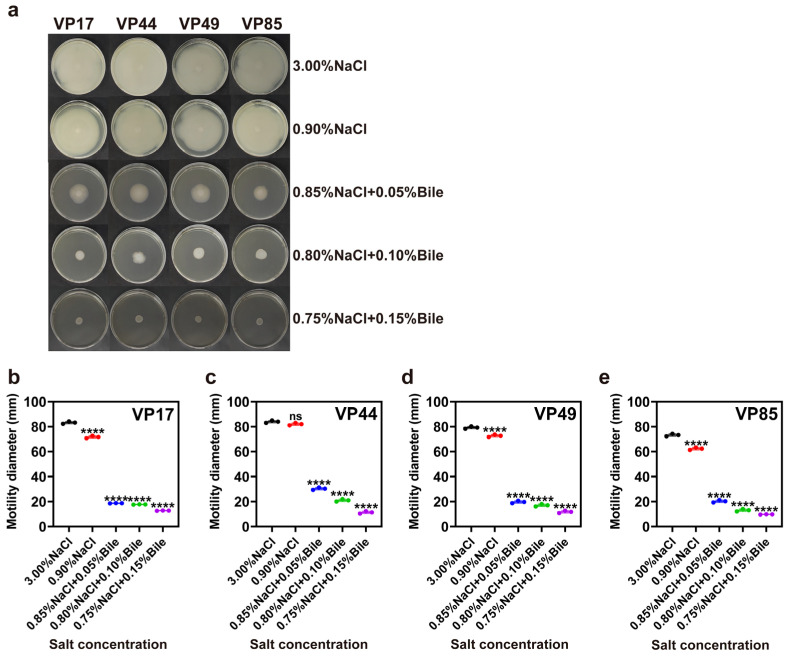
Impact of low salinity and bile salts on the motility of *V. parahaemolyticus.* (**a**) Swimming motility of four strains on plates with different salinities. (**b**–**e**) Motility diameter of VP17, VP44, VP49, and VP85 on swimming plates with different salinities. Significance was compared to the control, ns > 0.05, **** *p* < 0.0001.

Motility enhances the bacterial tolerance to adverse environments and plays a crucial role in infections and pathogenicity. Motility defects can reduce the ability of bacteria to invade hosts. This process is complex, closely related to colony species and growth environment, and exhibits diverse expression patterns. Specifically, flagellin expression, intracellular c-di-GMP levels, and the synthesis of quorum-sensing signaling molecules all affect swarming motility and collectively regulate bacterial behavior in colonies [12]. The results showed that the motility of VP17, VP44, VP49, and VP85 was affected when salinity was reduced to 0.90%. Meanwhile, the motility of all four strains was significantly inhibited under the dual stress of low salinity and bile salts (Figure 2).

### 3.3. Biofilm Membrane Production

Biofilms are communities of cells encased in extracellular polymeric substances (EPSs) secreted by microorganisms, forming complex three-dimensional structures attached to various surfaces [24]. Bacterial biofilm formation is closely related to their pathogenicity. Biofilm formation enables bacteria to persist in the host, resisting immune attack and causing chronic infections. It also limits the penetration of bactericidal substances (e.g., antibiotics), enhances bacterial tolerance to adverse environments, prevents bacterial clearance, and may lead to recurrent infections [25].

**Figure 3:**
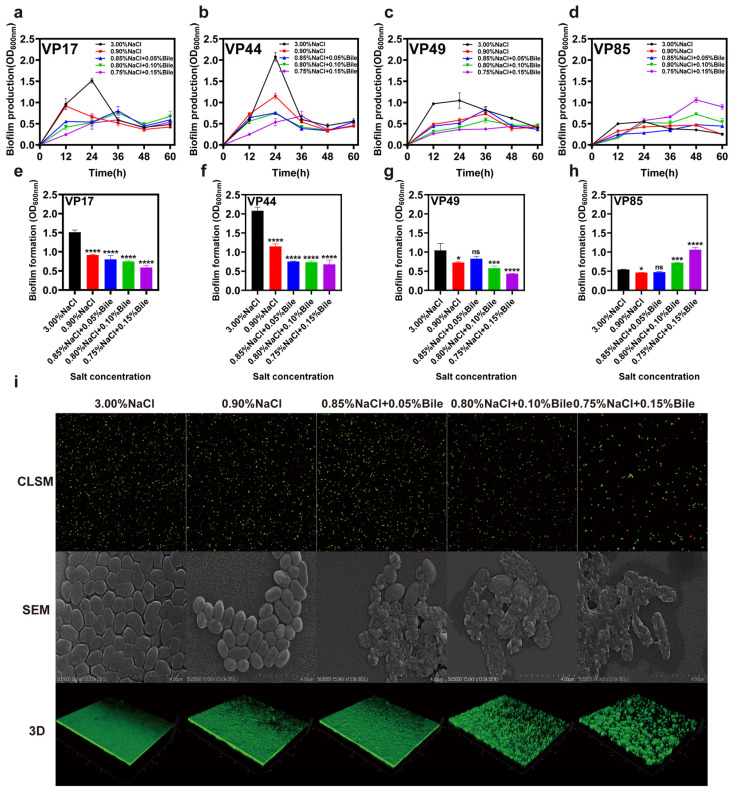
Impact of low salinity and bile salts on the biofilm of *V. parahaemolyticus.* (**a**–**d**) Comparison of biofilm production (OD_600nm_) of VP17, VP44, VP49, and VP85 over time under different salinities. (**e**–**h**) Maximum biofilm formation (max BF formation) of VP17, VP44, VP49, and VP85 under different salinities. (**i**) Viability, membrane morphology, and 3D CLSM images of VP17 under different salinities. CLSM stands for confocal laser scanning microscope and SEM stands for scanning electron microscopy. Scale bar: 4 μm. Significance was compared to the control, ns > 0.05, * *p* < 0.05, *** *p* < 0.001, **** *p* < 0.0001.

Figure 3b shows that VP44, which lacks virulence genes, exhibited no change in maximum biofilm production time under reduced salinity and low bile salts concentration, but was delayed to 36 h under high bile salts concentration. The VP17 strain, which only contains *tdh*, advanced its maximum biofilm production time from 24 h to 12 h under low salinity stress, rapidly forming biofilm to resist this stress. After bile salts were added, the maximum biofilm production was delayed to 36 h (Figure 3a). The VP49 strain, which contains *tdh* and *trh*, had its maximum biofilm production time delayed from 24 h to 36 h under both low salinity stress and bile salts stimulation (Figure 3c). Meanwhile, the maximum biofilm formation time of VP85, which contains *trh*, was 48 h under all culture conditions (Figure 3d). Thus, the dual stress of low salinity and bile salts more strongly inhibited biofilm formation in *V. parahaemolyticus* strains containing the virulence gene *trh* than in strains lacking this gene. Interestingly, under environmental stress, maximum biofilm formation (max BF formation) was significantly inhibited in VP17, VP44, and VP49 but enhanced in VP85, which contains *trh*. These findings warrant further investigation.

Additionally, we conducted viability analysis, cell membrane surface analysis, and biofilm structure analysis on VP17 (*tdh^+^*/*trh^−^*) cultured in media with different salinities. The results showed that when salinity decreased, the periplasm became smooth and rough. After the addition of bile salts, the bacterial cell membrane was destroyed, surface roughness increased or even cracked, and the periplasm became sparser. However, the strains did not die in large numbers as bile salts concentration increased (Figure 3i).

### 3.4. ROS Levels

**Figure 4:**
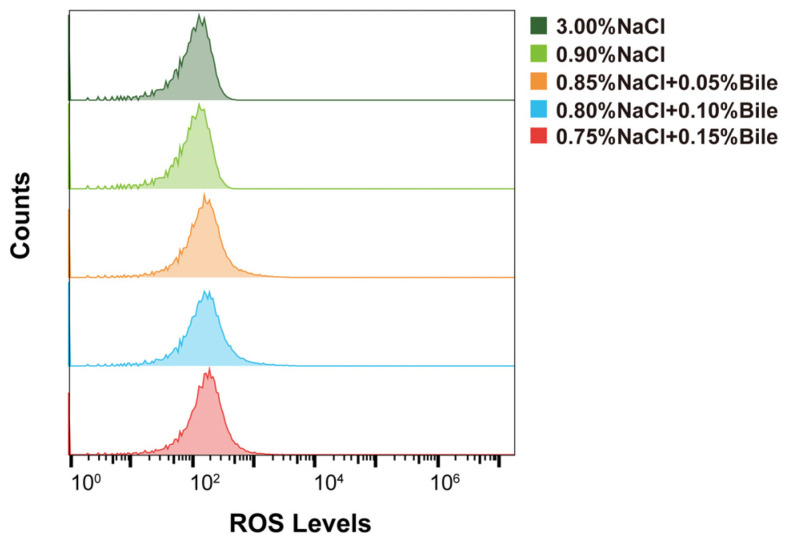
Intracellular ROS levels in VP17 under different salinity stresses.

Under bile salts stress, damage to membrane proteins, protein misfolding, and the inhibition of enzymes involved in electron transport, redox, and metabolic processes can increase oxidative stress and promote ROS generation [26]. Therefore, we measured the intracellular ROS levels in strains cultured under different salinity concentrations. The results showed that intracellular ROS levels in *V. parahaemolyticus* did not change after treatment with different concentrations of bile salts and low salinity (Figure 4).

### 3.5. Inhibition Rate of Cell

**Figure 5:**
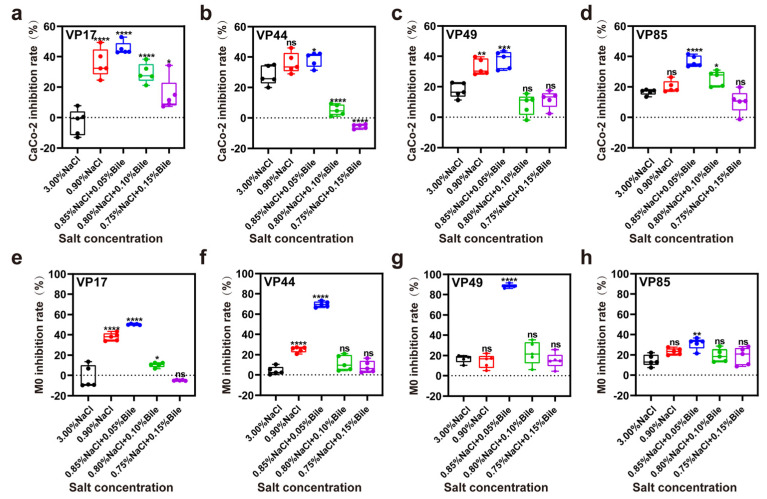
Inhibition of intestinal cells by virulence factors secreted by *V. parahaemolyticus* under different salinity stresses. (**a**–**d**) Inhibition rates of VP17, VP44, VP49, and VP85 on Caco-2 cells under different salinity stresses. (**e**–**h**) Inhibition rates of VP17, VP44, VP49, and VP85 on M0 cells under different salinity stresses. Significance was compared to the control, ns > 0.05, * *p* < 0.05, ** *p* < 0.01, *** *p* < 0.001, **** *p* < 0.0001.

Bile salts can stimulate the secretion of virulence factors by *V. parahaemolyticus*. Caco-2 and M0 were used as models to investigate the virulence of *V. parahaemolyticus* under low salinity and bile salts stress in the human intestine. The results showed that under low salinity stress, all four strains secreted low levels of virulence factors, killing Caco-2 cells. Toxicity to the cells was highest at low bile salts concentrations (0.05%) and decreased with increasing bile salts concentrations (Figure 5a–d). Similarly, M0 macrophages exhibited the same trend (Figure 5e–h). Consequently, *V. parahaemolyticus* secretes few virulence factors in pure culture but responds to increased stress by activating self-protection mechanisms and secreting virulence factors. Previous studies on *V. parahaemolyticus* enterotoxicity focused on T3SS activation by bile salts, but our results show that it also exhibits cytotoxicity under low salinity stress. Thus, low salinity stress is as important as bile salts stress when studying the pathogenic mechanisms of *V. parahaemolyticus*.

### 3.6. Proteomic Analysis of V. parahaemolyticus Under Dual Stress of Low Salinity and Bile Salts

#### 3.6.1. PCA Analysis of *V. parahaemolyticus* Protein Data Under Different Salinity Stresses

**Figure 6:**
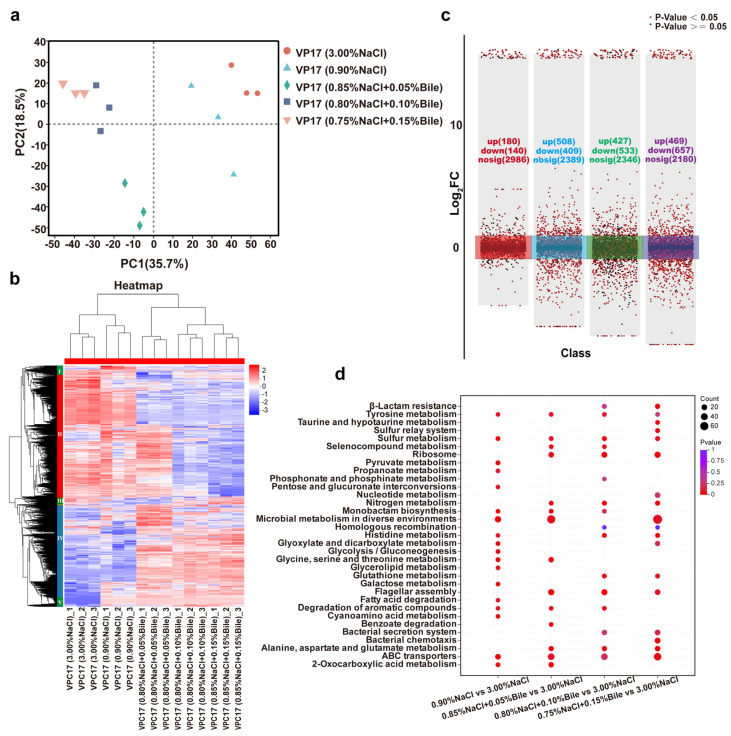
Proteomic data of *V. parahaemolyticus* under different salinity stresses. (**a**) PCA analysis of proteomics data. (**b**) Cluster heatmap of significant proteins in five samples. (**c**) Multiple volcano plots (red, blue, green, and purple, respectively, represent the comparison of protein expression levels between the experimental groups: 0.90% NaCl (*w*/*v*), 0.85% NaCl + 0.05% Bile (*w*/*v*), 0.80% NaCl + 0.10% Bile (*w*/*v*), 0.75% NaCl (*w*/*v*) + 0.15% Bile, and the control group 3.00% NaCl). (**d**) Comparison of enriched pathways between each experimental groups and the control group.

Previously, we analyzed the phenotypes of *V. parahaemolyticus* under different salinity treatments, including growth, motility, biofilm formation, etc. To further understand the phenotypic heterogeneity of *V. parahaemolyticus* under early human gut stress, we performed the proteomic analysis of VP17 under different salinity conditions, revealing the molecular mechanisms of its response and tolerance. The PCA results showed good intra-group reproducibility and a correlation between the two bile salt-free samples, as well as a strong correlation among the three bile salt-treated samples. These results indicate significant differences between the experimental and control groups, highlighting the importance of this study (Figure 6a).

#### 3.6.2. Differential Expression Protein Analysis

Multi-component protein clustering analysis showed that protein expression patterns were consistent within each group, indicating stable expression and good intra-group reproducibility. Moreover, cluster analysis revealed significant differences in protein expression patterns between the experimental and control groups. Proteins were classified into five groups based on their expression profiles. In the group I region, VPC17 protein was partially upregulated under low salinity stress and significantly downregulated under bile salts stress, and interestingly, the opposite was true for the group IV region and group I region. Under high bile salts stress, group II was significantly downregulated, whereas groups IV and V were activated and significantly upregulated across all experimental groups. These groups merit further investigation (Figure 6b).

Multiple volcano plots identified a total of 3306 proteins in this experiment. The sequencing data of VP17 cultured in LB medium containing 3.00% NaCl served as the control for downstream analysis. Differentially expressed proteins (DEPs) were identified under the conditions of |log2FC| ≥ 2 and *Padj* < 0.01. Compared with the LB medium containing 3.00% NaCl, the number of DEPs in media with 0.90% NaCl (*w*/*v*), 0.85% NaCl + 0.05% Bile (*w*/*v*), 0.80% NaCl + 0.10% Bile (*w*/*v*), and 0.75% NaCl (*w*/*v*) + 0.15% Bile were 320, 917, 960, and 1126, respectively. The number of upregulated proteins increased with increasing bile salts concentration. Among these conditions, *V. parahaemolyticus* exhibited the highest number of upregulated proteins in response to 0.05% bile salts and the lowest number of downregulated proteins (Figure 6c).

#### 3.6.3. GO Annotation and KEGG Enrichment Results of DEPs

Continued GO analysis of DEPs revealed that they were categorized into 7 biological process (BP) terms, 2 cellular component (CC) terms, and 14 molecular function (MF) terms, with significant enrichment in catalytic activity, binding, and transporter activity. In addition to GO annotation, the KEGG pathway analysis was performed on key DEPs to elucidate the regulatory pathways significantly altered in *V. parahaemolyticus* under low salinity and bile salts stress. Based on the number of enriched proteins and *p*-values, the top 20 KEGG pathways enriched by DEPs in each experimental group were analyzed. DEPs were primarily enriched in energy metabolism pathways, including glycolysis/gluconeogenesis, pyruvate metabolism, pentose and glucuronate interconversions, glyoxylate and dicarboxylate metabolism, glycine/serine/threonine metabolism, propanoate metabolism, galactose metabolism, and fatty acid degradation, when salinity was reduced to 0.90% NaCl compared to the control group. Compared to the low salinity environment, DEPs were primarily enriched in nitrogen metabolism, alanine, aspartate, and glutamate metabolism; ribosome; and flagellar assembly after the addition of bile salts. As the bile salts concentration increased, DEPs were enriched in β-lactam resistance, glutathione metabolism, bacterial secretion systems, and chemotaxis (Figure 6d).

## 4. Discussion

### 4.1. Downregulation of Energy Metabolism-Related Proteins Inhibited the Growth of Strains

**Figure 7:**
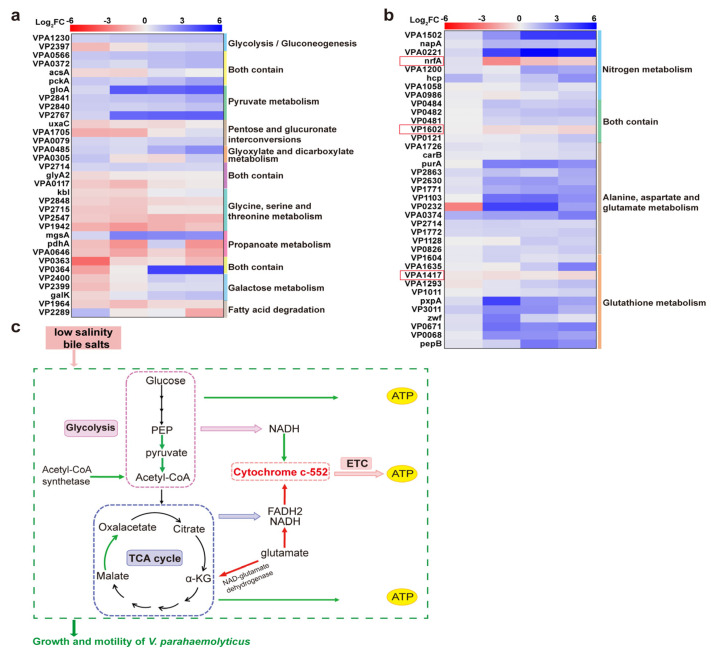
Impact of low salinity and bile salts on the energy metabolism of *V. parahaemolyticus.* (**a**,**b**) Heatmap of DEPs enriched in energy metabolism-related pathways of VP17 under different salinity stresses. From left to right is the comparison of protein expression levels between the experimental groups: 0.90% NaCl (*w*/*v*), 0.85% NaCl + 0.05% Bile (*w*/*v*), 0.80% NaCl + 0.10% Bile (*w*/*v*), 0.75% NaCl (*w*/*v*) + 0.15% Bile, and the control group 3.00% NaCl (*w*/*v*). (**c**) Schematic of energy metabolism pathways.

The KEGG pathway analysis revealed the downregulation of energy metabolism-related proteins under low salinity stress and bile salts stimulation (Figure 7a). Enzymes such as VPA1203, VPA0566, VPA0372, and pckA were downregulated, inhibiting pyruvate and acetyl-coenzyme A production. Additionally, malate oxidoreductase (VP2767) was downregulated, reducing oxaloacetate production and impeding the TCA cycle, thereby inhibiting energy production. Conversely, Acetyl-CoA synthetase expression was upregulated under low salinity and low bile salts concentration to promote Acetyl-CoA production and maintain energy metabolism. However, this effect was gradually inhibited as the bile salts concentration increased (Figure 7a,c). As bile salts concentration increased, most DEPs involved in nitrogen metabolism; alanine, aspartate, glutamate metabolism; and glutathione metabolism were downregulated, while cytochrome c-552 (nfrA), NAD-glutamate dehydrogenase (VPA1620), and glutathione S-transferase family protein (VPA1417) were upregulated. The upregulation of NAD-glutamate dehydrogenase promoted glutamate oxidation to α-ketoglutarate, ammonia, and NADH, providing TCA cycle substrates and enhancing energy metabolism. Cytochrome c-552 expression was upregulated, serving as a key component of the electron transport chain. This promoted the involvement of crucial electron carriers (e.g., NADH and FADH2) in oxidative phosphorylation, generating the ATP needed for bacterial survival (Figure 7b,c).

Bacteria adapt to environmental conditions through energy metabolism pathways, such as glycolysis, the TCA cycle, and ETC. However, these pathways were inhibited by low salinity and bile salts due to the suppression of related proteins. Consequently, as shown in Figure 1, the growth of the strains was significantly inhibited after environmental changes, with adaptation occurring through increased lag time (LT). Although halophilic *V. parahaemolyticus* can adapt to high-salinity environments, maintaining osmotic pressure balance requires energy [11]. As a result, strains cultured at 3.00% NaCl exhibited lower *μ_max_* values than those cultured at 0.90% NaCl. Conversely, after the addition of bile salts, their bactericidal properties significantly inhibited *V. parahaemolyticus* energy metabolism, extending LT and reducing *μ_max_*. We hypothesize that the upregulation of glutathione S-transferase family proteins, which transfer glutathione, helps eliminate ROS within bacteria, thereby reducing oxidative stress damage (Figure 4).

### 4.2. Upregulation of Ribosome-Associated Proteins to Synthesise Cell Membrane Repair-Associated Proteins

**Figure 8:**
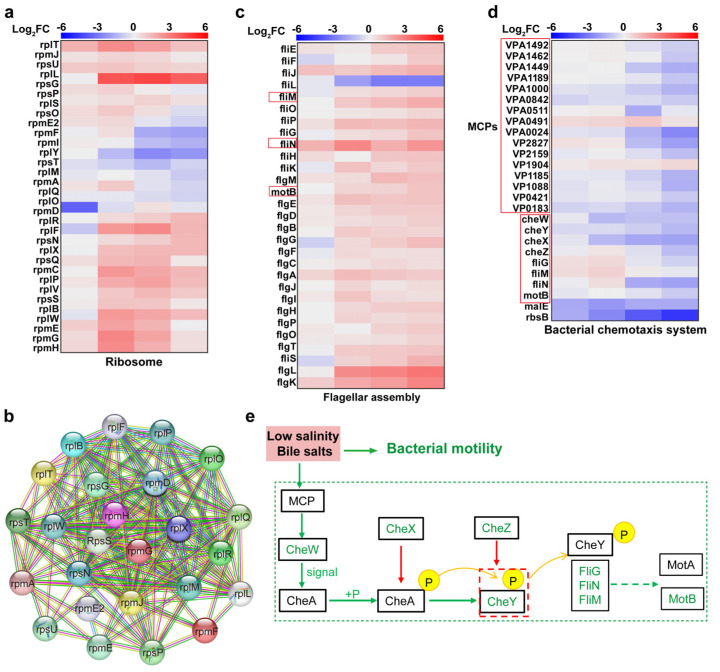
Impact of low salinity and bile salts on ribosomal, motility, and chemotaxis systems of *V. parahaemolyticus.* (**a**) Heatmap of DEPs enriched in VP17 ribosomal pathways under different salinity stresses. (**b**) Protein–protein interaction network diagram for ribosome-associated proteins. (**c**,**d**) Heatmap of DEPs enriched in VP17 motility and chemotaxis systems pathways under different salinity stresses. (**e**) Molecular mechanism diagram of *V. parahaemolyticus* motility inhibition under low salinity and bile salts stress. The heatmap from left to right is the comparison of protein expression levels between the experimental groups: 0.90% NaCl (*w*/*v*), 0.85% NaCl + 0.05% Bile (*w*/*v*), 0.80% NaCl + 0.10% Bile (*w*/*v*), 0.75% NaCl (*w*/*v*) + 0.15% Bile, and the control group 3.00% NaCl (*w*/*v*).

Bile salts, an antibacterial agent, can disrupt bacterial cell membranes, causing intracellular protein misfolding and resulting in DNA or RNA damage [27]. Ribosomes, as the “factory” for proteins that constitute more than half of the cellular biomass, significantly influence bacterial life activities through their expression levels [28]. Figure 8c shows that *V. parahaemolyticus* exhibited a significant enrichment of DEPs in the ribosomal pathway after bile salts stimulation, primarily involving large (RPL) and small (RPS) ribosomal subunit proteins, with strong protein–protein interactions (Figure 8a,b). Proteins of the RPL and RPS families are crucial components of ribosomes, forming the ribosome’s fundamental structure alongside rRNA. For example, rpmA is involved in ribosome assembly and is crucial for maintaining ribosome structural integrity [29]. Together with other ribosomal proteins and rRNA, it forms the functional core of the ribosome. Thus, under the dual stress of low salinity and bile salts, *V. parahaemolyticus* upregulated RPL and RPS expression, synthesizing the proteins required for normal life activities and membrane repair, resisting bile salts-induced membrane damage, and correcting protein misfolding to maintain protein homeostasis.

### 4.3. Downregulation of Chemotactic System and Flagellar Motor Proteins Inhibited V. parahaemolyticus Motility

Previous experiments showed that *V. parahaemolyticus* motility was significantly inhibited under low salinity and bile salts stress. Additionally, DEPs in various treatment groups were significantly enriched in the flagellar assembly and chemotaxis pathways, with enrichment increasing as the bile salts concentration rose. Methyl chemotactic proteins (MCPs), bacterial cell membrane receptor proteins, contain methylation sites that detect environmental changes and transmit signals to CheA via CheW. CheA is activated and autophosphorylated to CheA-P, which transfers the phosphate group to CheY, forming CheY-P. CheX inhibits CheA autophosphorylation, while CheZ promotes CheY-P dephosphorylation, accelerating CheY recycling. The downregulation of MCPs-related proteins impaired signal transmission to downstream receptors, while downregulation of CheX and CheZ increased CheY-P production. CheY-P binds to the N-terminal domains of the FliM, FliG, and FliN complex, determining flagellar rotation direction [30]. Thus, upregulated CheY-P expression maintains bacterial motility direction. As shown in Figure 8, although *V. parahaemolyticus* motility was significantly inhibited, the motility direction remained relatively uniform. FliG binds to MotA, connecting the rotor and stator. The stator contains transmembrane ion channels, and the potential difference generated by H^+^ or Na^+^ transmembrane transport drives stator–rotor interaction, producing flagellar torque and propelling bacterial motility [31]. Thus, the downregulation of FliG and MotA expression inhibited *V. parahaemolyticus* motility.

### 4.4. Regulation of Secretion System-Related Proteins Affected V. parahaemolyticus Virulence

The bacterial secretion system transports proteins from the intracellular to the extracellular or periplasmic space, including enzymes, toxins, and signaling molecules [32]. The type II secretion system (T2SS) is a significant protein secretion mechanism in Gram-negative bacteria. This two-step process transports unfolded proteins (polypeptides) to the periplasmic space via the Sec/Tat pathway, allowing complete folding, and then secretes them into the extracellular environment through the T2SS [33]. Under low salinity and bile salts stress, *V. parahaemolyticus* upregulated SecDF in the Sec pathway, enhancing SecYEG complex activity and promoting efficient protein transport. It also upregulated TatBC, a membrane protein receptor complex in the Tat pathway, which recognizes and binds substrate protein signal peptides, promoting correct targeting. The upregulation of GspM, GspL, GspK, GspJ, GspI, and GspG ensured normal T2SS assembly and function, promoting substrate secretion (e.g., toxins, enzymes). Together, the T2SS and Sec/Tat pathway promoted toxin secretion by *V. parahaemolyticus*, enhancing its virulence.

The type III secretion system (T3SS) is a key virulence factor of *V. parahaemolyticus*. This is essential for bacterial colonization and pathogenicity by delivering effector proteins to the host cell cytoplasm, thereby manipulating host cell functions [34]. The upregulation of ATPase (VscN) in T3SS hydrolyzes ATP to drive effector protein secretion from the bacterial cytoplasm into the host cell, enhancing virulence. However, the expression of T6SS-related proteins (icmF, clpV) was inhibited. Consequently, under low salinity and bile salts stress, T6SS exerted minimal toxicity, while T2SS and T3SS primarily mediated effector protein secretion to enhance virulence.

### 4.5. Activation of the Beta-Lactam Resistance Pathway Promoted Bile Salts Efflux to Reduce Cytotoxicity

**Figure 9:**
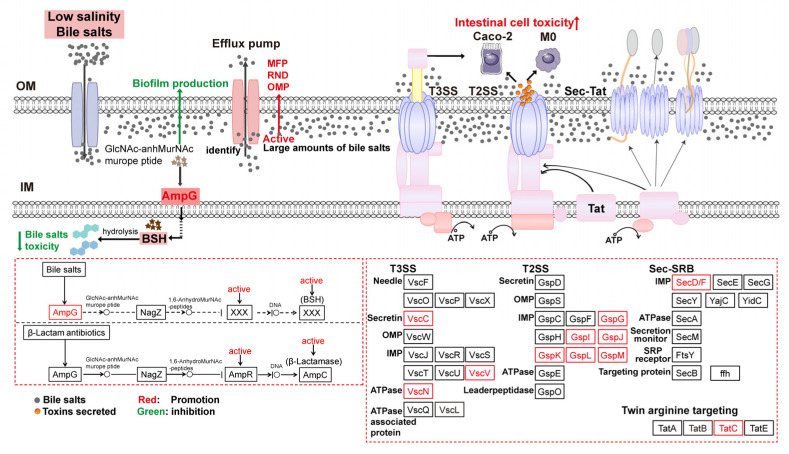
Tolerance mechanisms of *V. parahaemolyticus* under low salinity and high bile salts concentration.

DEPs were enriched in the beta-lactam resistance pathway under low salinity stress when bile salts concentration increased. Among them, AmpG, a membrane channel protein, detects extracellular peptidoglycan alterations and transports GlcNAc-anhMurNAcmuropeptides into the cytoplasm. Accumulated AmpR activates the expression of AmpC enzyme (β-lactamase), which hydrolyzes β-lactam antibiotics, rendering them inactive and enhancing bacterial resistance [35]. Under low salinity and bile salts stress, cell membrane damage in *V. parahaemolyticus* produces GlcNAc-anhMurNAcmuropeptides. The upregulation of AmpG promotes the conversion of these peptides into bile salts hydrolases (BSH), which catalyze the hydrolysis of glycine or taurine residues to produce de-conjugated bile salts. This reduces toxicity and improves strain tolerance to bile salts (Figure 9). Meanwhile, mrdA and ftsI encode PBP2 and PBP3, respectively, which are involved in cell wall peptidoglycan synthesis. Their upregulation maintains cell wall extension and morphology.

The activation of β-lactam resistance enhances bacterial tolerance to bile salts but is detrimental when large amounts enter cells. Efflux pumps are membrane transport proteins that reduce the intracellular concentrations of xenobiotics (e.g., antibiotics, toxins) and thereby mitigate their detrimental effects. Four main types of drug efflux pump systems are known: the major facilitator superfamily (MFS), small multidrug resistance (SMR) family, resistance-nod-cell division (RND) family, and ATP-binding cassette (ABC) superfamily [36]. Analysis of enriched efflux pump proteins revealed that the expression of OMP-, RND-, and MFP-related proteins was upregulated in *V. parahaemolyticus* under low salinity and bile salts stress, enhancing the export of bile salts.

**Figure 10:**
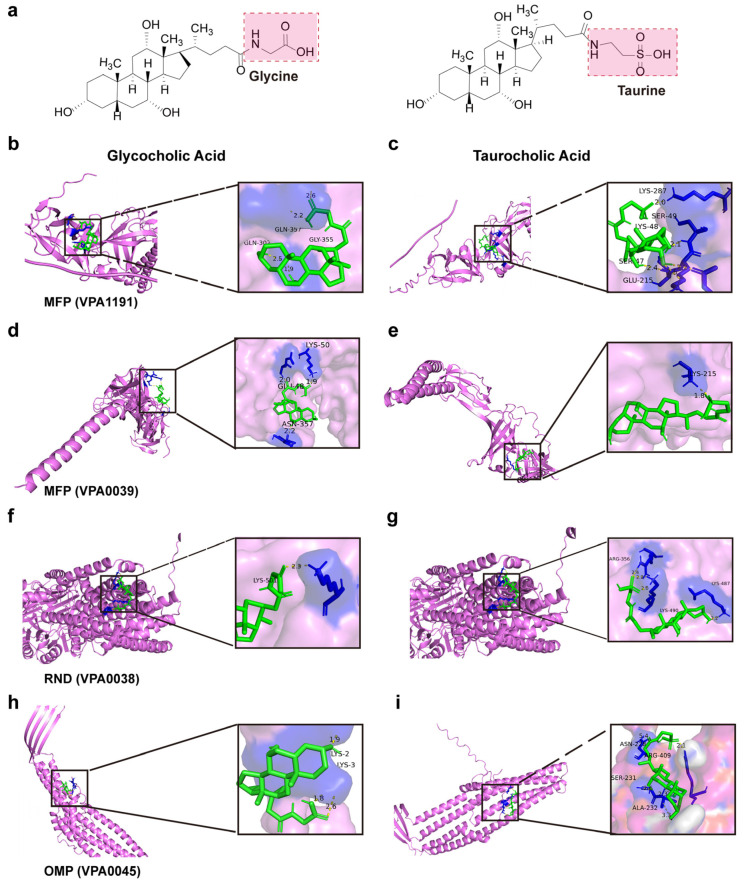
Molecular docking diagrams of efflux pump proteins with bile salts molecules. (**a**) Bile salts molecular structure. (**b**,**c**) Molecular docking of bile salts with MFP (VPA1191). (**d**,**e**) Molecular docking of bile salts with MFP (VP0039). (**f**,**g**) Molecular docking of bile salts with RND (VP0038). (**h**,**i**) Molecular docking of bile salts with OMP (VP0425).

Investigating interactions between antimicrobial compounds and efflux pump proteins provides comprehensive insights into *V. parahaemolyticus* bile salts tolerance, including binding sites, modes, and energy changes. Molecular docking diagrams show the binding modes between efflux pump proteins and bile salts molecules (Figure 10a–i). Bile salts interact with efflux pump proteins through hydrogen bonding, hydrophobic interactions, and conjugation, resulting in negative binding energy. Thus, efflux pump proteins more efficiently recognize and bind bile salts, facilitating their efflux and reducing intracellular bile salts concentration. Based on the binding pattern, amino acid residues at the binding sites between bile salts molecules and efflux pump proteins can be clearly identified (Table 2). Drugs that bind more efficiently to efflux pump proteins and inhibit their efflux function can be designed to reduce bacterial tolerance to antimicrobial substances. This provides a basis for developing new antibacterial drugs or efflux pump inhibitors against *V. parahaemolyticus*.

## 5. Conclusions

In summary, this study investigated the phenotypes of four strains of *V. parahaemolyticus* containing different virulence genes in the human intestinal tract under low salinity and bile salts stress. Proteomics was used to analyze their phenotypic heterogeneity and tolerance mechanisms, laying a theoretical foundation for understanding the pathogenic mechanisms of its sustained proliferation in the human body. Additionally, the results indicated that the β-lactam resistance pathway was a key pathway under low salinity and bile salts stress and played an important role in bile salts resistance. Overall, this study provides a foundational framework for further exploration into the pathogenic mechanisms of halophilic *V. parahaemolyticus* under low salinity conditions relevant to human physiology. Particularly, the binding site of the efflux pump protein activated by bile salt stress in *V. parahaemolyticus* represents a potential target for the development of novel antimicrobial therapeutics.

## Figures and Tables

**Table 1:** Four strains of *V. parahaemolyticus* and cell lines.

Strains/Cell Lines	*trh*	*tdh*	*tlh*	Source
VP17	−	+	+	Fecal specimens with acute diarrhea
VP44	−	−	+	Fecal specimens with acute diarrhea
VP49	+	+	+	Fecal specimens with acute diarrhea
VP85	+	−	+	Fecal specimens with acute diarrhea
THP-1				Primary cells purchased from Beyotime Biotechnology
Caco-2				Primary cells purchased from Beyotime Biotechnology

“+” means positive; “−” means negative. *trh*, *tdh*, and *tlh* are common virulence genes in *V. parahaemolyticus*.

**Table 2:** Docking of bile salts molecules to efflux pump proteins score.

Efflux Pump Proteins	Bile Salts Molecule	Mole Dock Score (Kcal/mol)	Interaction Residues
MFP (VPA1191)	Glycocholic acid	−4.99	Gln357, Gln307, and Gly355
MFP (VPA1191)	Taurocholic acid	−2.92	Lys48, Lys287, Ser47
MFP (VP0039)	Glycocholic acid	−4.55	Glu48, Lys50, and Asn357
MFP (VP0039)	Taurocholic acid	−3.78	Lys215
RND (VP0038)	Glycocholic acid	−5.15	Lys501
RND (VP0038)	Taurocholic acid	−3.69	Lys487, Lys490, Arg356
OMP (VP0425)	Glycocholic acid	−4.37	Lys2, Lys3
OMP (VP0425)	Taurocholic acid	−4.33	Asn228, Arg409, Ser2

## Data Availability

Data will be made available on request.
